# Hippocampal microscopic fractional anisotropy is reduced in temporal lobe epilepsy

**DOI:** 10.1162/imag_a_00356

**Published:** 2024-11-07

**Authors:** Nico J.J. Arezza, Hana H. Abbas, Caroline Chadwick, Ingrid S. Johnsrude, Jorge G. Burneo, Ana Suller Marti, Ali R. Khan, Corey A. Baron

**Affiliations:** Department of Medical Biophysics, Schulich School of Medicine and Dentistry, Western University, London, ON, Canada; Robarts Research Institute, Schulich School of Medicine and Dentistry, Western University, London, ON, Canada; Department of Psychology, Western University, London, ON, Canada; Epilepsy Program, Schulich School of Medicine and Dentistry, Western University, London, ON, Canada; Neuroepidemiology Unit, Schulich School of Medicine and Dentistry, Western University, London, ON, Canada; Pediatric Department, Schulich School of Medicine and Dentistry, Western University, London, ON, Canada

**Keywords:** temporal lobe epilepsy, hippocampus, cornu ammonis, microscopic fractional anisotropy, diffusion MRI, hippUnfold

## Abstract

Surgical resection is the method of choice for treating drug-resistant focal temporal lobe epilepsy (TLE). Postsurgical outcomes are better when magnetic resonance imaging (MRI) findings can localize the seizure focus for resection. However, many patients are MR-negative, meaning the focus cannot be differentiated from normal tissue in relaxation-weighted MRI. Diffusion MRI shows promise as a preoperative marker of neuronal abnormalities due to its sensitivity to cellular changes such as axon damage, indexed by fractional anisotropy. Microscopic fractional anisotropy is a recently introduced diffusion MRI metric that is sensitive to axon integrity regardless of axon orientation in both gray and white matter. In contrast, regular fractional anisotropy is only sensitive to axon integrity in coherently oriented bundles of fibers. This work investigated whether microscopic fractional anisotropy is sensitive to hippocampal abnormalities in drug-resistant TLE. Diffusion MRI was performed on a 3T scanner in 19 patients (age = 31 ± 10 years) with drug-resistant TLE (of which 10 were MR-negative) and 18 healthy volunteers (age = 38 ± 15). A deep-learning method was employed to segment the hippocampus into smaller subregions corresponding to the subiculum, cornu ammonis (CA) 1, CA2/3, and CA4 plus dentate gyrus (DG). Mean measurements of subregion volume, diffusivity, fractional anisotropy, and microscopic fractional anisotropy were compared between cohorts. In a subset of the TLE cohort suspected to have unilateral pathology (n = 15, age = 32 ± 10 years), the percentage differences between measurements ipsilateral and contralateral to the epileptogenic zone were evaluated to assess asymmetry. Microscopic fractional anisotropy was reduced in the hippocampus of drug-resistant TLE patients relative to healthy volunteers. In subregion-specific analysis, microscopic fractional anisotropy was significantly reduced in only the CA4/DG region in patients compared with healthy volunteers, after corrections for multiple comparisons. In the 15 patients with suspected unilateral pathology, microscopic fractional anisotropy was reliably and statistically lower in the ipsilateral CA4/DG region than in the contralateral side. Significant differences were not observed between TLE patients and healthy volunteers, or between hemispheres for patients with suspected unilateral pathology, for the fractional anisotropy or volume metrics. Diffusion MRI may complement standard imaging procedures by detecting abnormalities in MRI-negative patients. Due to its ability to detect abnormality regardless of axon orientation, microscopic fractional anisotropy may improve seizure focus localization in surgical candidates.

## 1 Introduction

Temporal lobe epilepsy (TLE) is the most common form of focal epilepsy in adults, with as many as two-thirds of seizure foci being localized to the temporal lobe (Bell et al., 2009;Engel, 2006;Georgiadis et al., 2013). Although antiseizure medications provide seizure relief for many patients, approximately 30% of adults with epilepsy have seizures that do not respond to epilepsy medications (Georgiadis et al., 2013;Kwan & Brodie, 2000). Surgical resection of the seizure focus can be an effective treatment for drug-resistant TLE (Vadera et al., 2012;Wiebe et al., 2001). The hippocampus is the most common epileptogenic zone in TLE (Blumcke et al., 2017;Cascino et al., 1991;Falconer & Serafetinides, 1963), and hippocampal sclerosis, which manifests as signal change and atrophy of the hippocampus detected by MRI (Chabardès et al., 2005), is the best predictor of seizure freedom in patients who undergo resective surgery (Falconer & Serafetinides, 1963;Wieser & ILAE Commission on Neurosurgery of Epilepsy, 2004). Seizure freedom following surgical resection is achieved in 75% of patients with clearly delineated HS in preoperative MRI (i.e., MR-positive or MR+ patients), but in only 51% of MR-negative patients (Muhlhofer et al., 2017), perhaps because the seizure focus has not been adequately localized and/or the resection does not include the entire epileptogenic zone. This demonstrates the need for highly sensitive imaging techniques to complement the current standard MRI, prolonged video-EEG, neuropsychological assessment, and nuclear medicine techniques, to improve seizure focus localization.

The International League Against Epilepsy (ILAE) outlines three histopathological HS subtypes (Blümcke et al., 2013): Type 1 is characterized by severe neuron loss and gliosis primarily in the cornu ammonis (CA) 1 and 4 hippocampal subregions; in Type 2, cell loss and gliosis predominately affect the CA1 region; and in Type 3, they predominately affect CA4. Hemispheric volume asymmetry of the entire hippocampal formation is a clinical standard for MRI-based detection of HS (Bernasconi et al., 2019), but does not consider subregion-specific pathology. Furthermore, hemispheric volume asymmetry is not necessarily indicative of pathology or injury, as it can result from left- or right-side dominance or congenital differences between the hemispheres (Woolard & Heckers, 2012). Imaging techniques that can detect HS with sufficient resolution to determine the HS subtype, and with greater specificity to pathology than volume measurements, could potentially improve surgical outcomes by better localizing the seizure focus.

Diffusion-weighted MRI (dMRI) is a promising technique for visualizing tissue pathology in epilepsy due to its sensitivity to neuron microstructure (Lorio et al., 2020). The diffusion tensor imaging (DTI) parameters fractional anisotropy (FA) and mean diffusivity (MD) are of particular interest because demyelination, reduced axon density, and widened extracellular spaces due to gliosis reduce FA and increase MD (Winston et al., 2020). Previous studies have shown that reduced FA and increased MD are apparent in various brain regions in TLE patients (Muhlhofer et al., 2017;Scanlon et al., 2013;Winston et al., 2020), and that increased MD is apparent in the ipsilateral hippocampus in patients with unilateral TLE (Goubran et al., 2016;Treit et al., 2019). Despite these results, the interpretation of FA being a surrogate marker of axon density is impaired in regions with crossing or fanning neuron-fibers because it is sensitive to both microstructure and intravoxel fiber orientation dispersion (Jones et al., 2013;Tournier et al., 2011). Reduced FA measurements are obtained in regions with complex fiber orientations (Oouchi et al., 2007); thus, FA cannot reliably index tissue pathology in TLE because the hippocampus contains crossing-fiber regions (Sato et al., 2017).

Microscopic fractional anisotropy (μFA) is a dMRI metric that quantifies water diffusion anisotropy independent of both neuron-fiber orientation dispersion and compartment size (Lasič et al., 2014;Shemesh et al., 2016;Westin et al., 2016) with high reproducibility and low bias when acquired with clinical MRI systems (Arezza et al., 2021;Kerkelä et al., 2021;Szczepankiewicz et al., 2019). Generally, μFA can distinguish between anisotropy resulting from microstructure and anisotropy resulting from axon orientation by exploiting the contrast between two different dMRI acquisitions (Cory et al., 1990;Lasič et al., 2014;Shemesh et al., 2016;Szczepankiewicz et al., 2015): (1) acquisitions that each probe diffusion in a single direction (i.e., encoding that is typically used in dMRI) and (2) acquisitions that probe diffusion in multiple orthogonal directions simultaneously, called “b-tensor encoding.” Previous studies have demonstrated that μFA outperforms FA for delineating lesions in multiple sclerosis (Yang et al., 2018), for evaluating white matter degeneration in Parkinson’s disease (Ikenouchi et al., 2020), and for distinguishing between different types of brain tumors (Szczepankiewicz et al., 2015), among other potential applications. In the TLE clinical workflow, μFA may provide a complementary metric to the current imaging and EEG techniques due to its sensitivity to microstructure and insensitivity to fiber orientation, particularly in brain regions containing crossing fibers, such as the hippocampus (Yoo et al., 2022).

This work aimed to investigate the sensitivities of μFA, FA, MD, and regional volume to detect hippocampal pathology in patients with TLE. A deep-learning surface-based hippocampus unfolding pipeline called*HippUnfold*(DeKraker et al., 2022) was employed to segment the hippocampus into smaller subregions to isolate the specific regions that are affected in the HS ILAE subtypes. In patients with suspected unilateral disease, asymmetries in measurements of anisotropy, diffusivity, and volume between the ipsilateral and contralateral hemispheres may indicate pathology that can lateralize and help localize the seizure focus. We hypothesized that μFA may be more sensitive to hippocampal pathology than FA due to its independence from neuron-fiber orientation and may usefully complement the current standard of care for diagnostic or preoperative imaging in TLE.

## 2 Methods

### 2.1 Participants

Nineteen drug-resistant TLE patients (9 female and 10 male, mean age ± standard deviation = 31 ± 10 years) and 18 healthy volunteers (11 female and 7 male, mean age ± standard deviation = 38 ± 15 years) were included in this study, which was approved by the Health Sciences Research Ethics Board at Western University. Informed consent was obtained from all participants prior to their recruitment. The following inclusion criteria were used to determine eligibility for the TLE cohort: all patients (a) had a history of drug-resistant epilepsy, (b) underwent radiological and/or comprehensive scalp EEG assessments to identify and lateralize the epileptogenic region, (c) were suspected to have a seizure focus located in the temporal lobe, (d) had normal hearing and normal or corrected to normal vision, and (e) had basic proficiency in English. For the healthy control cohort (referred to herein as HC), the following inclusion criteria were applied: all volunteers (a) had normal hearing and normal or corrected to normal vision; (b) basic proficiency in English; and (c) had no history of neurological disorder, head injury, or psychoactive medication. Two volunteers were excluded after participation in the imaging protocol because they were taking psychoactive medications. Clinical and demographic information for the patient participants are shown inTable 1.

**Table 1.** Clinical characteristics of patients with temporal lobe epilepsy.

ID	Age	Sex	Handedness	Lateralization		HS in MRI
				EEG	PET	
**1**	**25–29**	**F**	**R**	**B**	**R**	**HS-**
2	40–44	M	R	L	L	L
3	55–59	M	R	L	N/A	L
4	20–24	F	R	L	L	HS-
5	20–24	M	R	R	N/A	HS-
6	30–34	F	R	L	L	HS-
7	30–34	F	R	L	L	L
8	35–39	F	R	R	N/A	HS-
**9**	**25–29**	**M**	**R**	**L**	**R**	**R**
**10**	**20–24**	**M**	**R**	**B**	**B**	**HS-**
11	25–29	M	R	R	N/A	R
12	20–24	M	R	L	B	L
13	25–29	M	L	R	R	HS-
14	25–29	M	R	L	L	HS-
15	25–29	F	R	R	N/A	HS-
**16**	**35–39**	**F**	**R**	**B**	**B**	**B**
17	30–34	F	R	L	L	HS-
18	40–45	F	A	L	L	B
19	35–39	M	R	L	L	HS-

The bolded cells indicate patients excluded from the suspected unilateral*TLE*subgroup because of suspected bilateral pathology.

EEG: Electroencephalogram, PET: Positron Emission Tomography, L: Left, R: Right, A: Ambidextrous, B: Bilateral, N/A: Not Available, HS: HS detected by clinical MRI (3T), HS-: HS not detected by clinical MRI (3T).

### 2.2 MRI acquisition and processing

Participants were scanned using a 3T full-body MRI system (Siemens Prisma) with a 32-channel head coil at the Centre for Functional and Metabolic Mapping at Western University. The protocol consisted of two anatomical MRI scans followed by two dMRI protocols for separate computation of DTI metrics and μFA. The first anatomical scan was a T1-weighted magnetization-prepared rapid acquisition with gradient echo (MPRAGE) sequence with repetition time/echo time (TE/TR) = 2.3/2400 ms and inversion time = 1.06 s, and the second anatomical scan was a T2-weighted sequence with TE/TR = 564/3200 ms. Both the T1- and T2-weighted scans had a field-of-view (FOV) = 240 x 256 mm^2^, 0.8 mm isotropic voxel size, and used rate 2 generalized autocalibrating partially parallel acquisitions (GRAPPA). The first dMRI protocol, for computing DTI metrics, used a multiband echo-planar imaging (EPI) sequence obtained from the Center for Magnetic Resonance Research (Setsompop et al., 2012) with TE/TR = 99/5500 ms, simultaneous multislice (SMS) factor 3, FOV = 222 x 222 mm^2^, and 1.6 mm isotropic voxel size to acquire 6, 36, and 60 linear tensor-encoded (LTE) volumes at b-values of 0, 1000, and 2000 s/mm^2^, respectively, and a b = 0 scan in the opposite phase-encoding direction, with a total scan time of 9 min. The second dMRI protocol, for the measurement of μFA, used a multiband EPI sequence (Siemens diffusion EPI sequence with diffusion gradients modified in-house) with TE/TR = 92/4900 ms, SMS factor 2, rate 2 GRAPPA, FOV = 229 x 229 mm^2^, and 1.8 mm isotropic voxel size to acquire 8 LTE volumes at b = 2000 s/mm^2^and 3, 6, and 16 spherical tensor-encoded (STE) volumes at b = 100, 1000, and 2000 s/mm^2^, respectively, with a total scan time of 3 min. The second protocol was performed twice, first with anterior-to-posterior and then with posterior-to-anterior phase encoding directions. Principal component analysis denoising and Gibbs’ ringing artifact correction were performed on the dMRI volumes with the*dwidenoise*(Veraart, Fieremans, et al., 2016;Veraart, Novikov, et al., 2016) and*mrdegibbs*(Kellner et al., 2016) tools from Mrtri x 3 (Tournier et al., 2019) and the data were then corrected for EPI readout and eddy current distortions using*topup*(Andersson et al., 2003) and*eddy*(Andersson & Sotiropoulos, 2016) from FSL 6.0 (Smith et al., 2004).

### 2.3 Hippocampus segmentation

*HippUnfold*v0.5.1 was used to segment the hippocampus into subiculum (SB), cornu ammonis (CA) 1–4, and dentate gyrus (DG) subregions, using the T2-weighted volume as input. The volumetric subregion segmentations from*HippUnfold*were used in this study, which are generated by (1) segmentation of hippocampal tissue and external boundaries with a U-net model, (2) mapping the intrinsic coordinates of the hippocampal gray matter using Laplace’s equation, and (3) transferring subregion boundaries to each individual tissue segmentation using the unfolded coordinates and a subregion atlas defined from a 3D histology reference space (labeling CA1, CA2, CA3, CA4, subiculum, and dentate gyrus). To reduce the number of comparisons during analysis, some of the subregions were combined to form distinct subregions based on the ILAE HS subtypes. Although significant cell loss is observed in CA2 and/or CA3 in some TLE patients, these findings are not consistent across any of the HS ILAE types (Blümcke et al., 2013), so these adjacent regions were combined into one subregion. The CA4 subregion was combined with the adjacent DG since cell loss scores in the DG tend to be higher in CA4-predominant HS type 1 and type 3 than in type 2 (Blümcke et al., 2013). The CA1 subregion was not merged with any others as it is of interest in HS type 1 and type 3.

### 2.4 Estimation of dMRI parameters

MD and FA maps were computed by fitting the dMRI data with b ≤ 1000 s/mm^2^from the first protocol to the DTI signal representation using a weighted linear least-squares method (Basser et al., 1994;Veraart et al., 2013). The LTE and STE data from the second protocol were jointly fitted to the powder average kurtosis signal representation (Jensen et al., 2005) to estimate μFA (Arezza et al., 2021;Lasič et al., 2014;Nilsson et al., 2020).

The T1-weighted image volumes were separately registered to the 1.8 mm resolution dMRI space and to the 1.6 mm resolution dMRI space, and then the inverse transformations were used to register the respective MD, FA, and μFA maps to the anatomical space. To ensure good registration quality, outlines of the hippocampal subregions were overlaid on top of the registered MD, FA, and μFA maps and were visually inspected.

### 2.5 Analysis of patients versus controls

For each drug-resistant TLE patient and healthy volunteer, the mean MD, FA, and μFA were measured in each of the four hippocampal subregions and full hippocampus, with each measurement spanning both hemispheres by averaging the left- and right-side measurements. The volumes of each subregion were measured by computing the sum of the number of voxels in each region in the T1-weighted image volume. To assess whether the diffusion metrics correlate with hippocampal volume, the Spearman’s rank correlation coefficient between relevant variables was computed for the full hippocampus, separately for each cohort. A correlation between any of the diffusion metrics and volume in the healthy cohort could indicate that the diffusion metric is modulated by the size of the hippocampus, potentially confounding changes resulting from microstructural pathology. To maximize the chance of detecting any such correlations, no corrections were made to account for multiple comparisons.

For each of the four subregions and the full hippocampus, Mann–Whitney U tests were performed to test for significant differences in measurements between the TLE and HC cohorts. To account for multiple comparisons, the Bonferroni correction was applied by dividing the starting p-value threshold of 0.05 by 16 (4 regions x 4 metrics) for subregion-specific analysis, and by 4 (4 metrics) for full hippocampus analysis (Dunn, 1961). It was hypothesized that volume, FA, and μFA may be reduced, and MD may be elevated, in some hippocampal regions in the TLE cohort relative to the healthy volunteers due to tissue atrophy, gliosis, and other changes to microstructure.

Measurements spanning both hemispheres may exhibit reduced sensitivity due to a lack of tissue pathology on one side in patients with unilateral disease. To compare the ability of single-hemisphere measurements versus measurements spanning both hemispheres in classifying TLE patients from healthy volunteers, first we identified the subregions in which statistically significant bilateral differences between the TLE and HC cohorts were observed in any of the metrics. Next, receiver operating characteristic (ROC) curves, which characterize the ability to distinguish patients from controls, were generated for each metric in those subregions under two different conditions: (1) measurements that spanned both hemispheres and (2) measurements made in a single hemisphere. For the single-hemisphere ROC curves, the hemisphere with the lower mean measurement was chosen for FA, and µFA, the hemisphere with the smaller hippocampus was chosen for volume, and the hemisphere with the greater mean measurement was chosen for MD, for each patient and healthy volunteer.

### 2.6 Analysis of left–right asymmetry

To assess each metric’s ability to correctly lateralize the seizure focus in patients with unilateral disease, a subgroup of the TLE cohort was isolated to include only those patients with suspected unilateral TLE. This subgroup was identified by selecting the patients whose EEG suggested unilateral (right or left) pathology. Three patients were excluded because their EEG suggested bilateral pathology, and a fourth patient was excluded out of caution because their MRI and PET both indicated right-side pathology, which contradicted the left-side pathology suggested by the patient’s EEG. After applying these criteria, 15 suspected unilateral TLE patients remained (7 female and 8 male, mean age ± standard deviation = 32 ± 10 years), of which 9 were MR-negative. The mean and standard deviation of each of the four measurements of interest, across all 15 patients, were separately calculated for each subregion ipsilateral and contralateral to the suspected pathology, and in the full hippocampus. In the healthy cohort, measurements of each subregion and of the whole hippocampus were made in both hemispheres. For the suspected unilateral TLE cohort only, Wilcoxon signed-rank tests were performed to test for significant differences between the ipsilateral and contralateral measurements, with the starting p-value threshold of 0.05 divided by 16 for subregion-specific analysis, and by 4 for full hippocampus analysis, to correct for multiple comparisons.

Hemisphere asymmetries in the volume, MD, FA, and μFA metrics in each region were computed in the TLE group using the following formula:

$$a=\frac{X_{contra}-X_{ipsi}}{0.5(X_{contra}+X_{ipsi})}*100\text{\%},$$

whereais an asymmetry index equal to the percentage difference between measurements ipsilateral and contralateral to the suspected pathology, andXis the mean subregion measurement. It was hypothesized that volume, FA, and μFA may be reduced, and MD may be elevated, in some ipsilateral regions relative to their respective contralateral counterparts due to tissue atrophy, gliosis, and changes to microstructure. Notably, asymmetries may be more likely to be observed in the CA1 and CA4/DG subregions that are predominantly affected in HS than in the SB and highly variable CA2/3 subregions. For the healthy group, absolute asymmetry ratios were computed to account for the lack of an ipsilateral/contralateral side distinction and to maximize asymmetry:

$$\left|a\right|=\left|\frac{X_{min}-X_{max}}{0.5(X_{max}+X_{min})}\right|*100\text{\%},$$

where$X_{max}$is the measurement from the hemisphere with the greater value and$X_{min}$is the measurement in the hemisphere with the lesser value. Maximizing asymmetry values renders comparison with values from patients with suspected unilateral pathology more conservative.

## 3 Results

Example sagittal and coronal T1- and T2-weighted images from one of the healthy volunteers are depicted inFigure 1with the four hippocampal subregions outlined. All hippocampal segmentations were manually inspected for accuracy in delineating the hippocampal tissue and subregions. Coronal slices of T1-weighted MRI, MD, FA, and μFA from a TLE patient with confirmed HS are depicted inFigure 2for comparison.

**Fig. 1.**
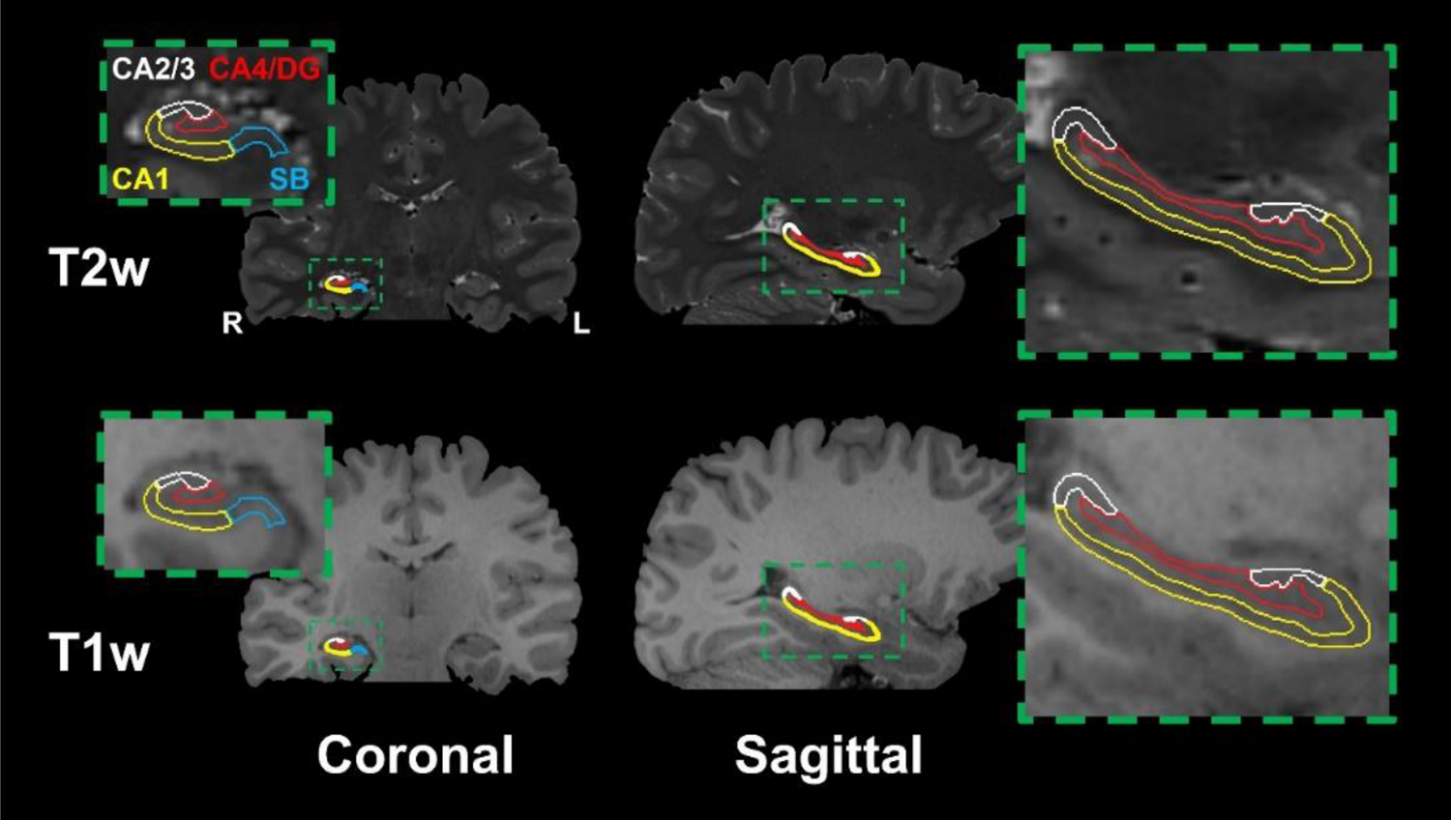
Coronal and sagittal T2-weighted (top) and T1-weighted (bottom) MR images from a healthy volunteer with insets highlighting the four hippocampal subregions used in this study: the subiculum (SB), cornu ammonis 1 (CA1), cornu ammonis 2 and 3 (CA2/3), and cornu ammonis 4 plus dentate gyrus (CA4/DG). Note that only the right hippocampus is labeled although both hippocampi were analyzed.

**Fig. 2.**
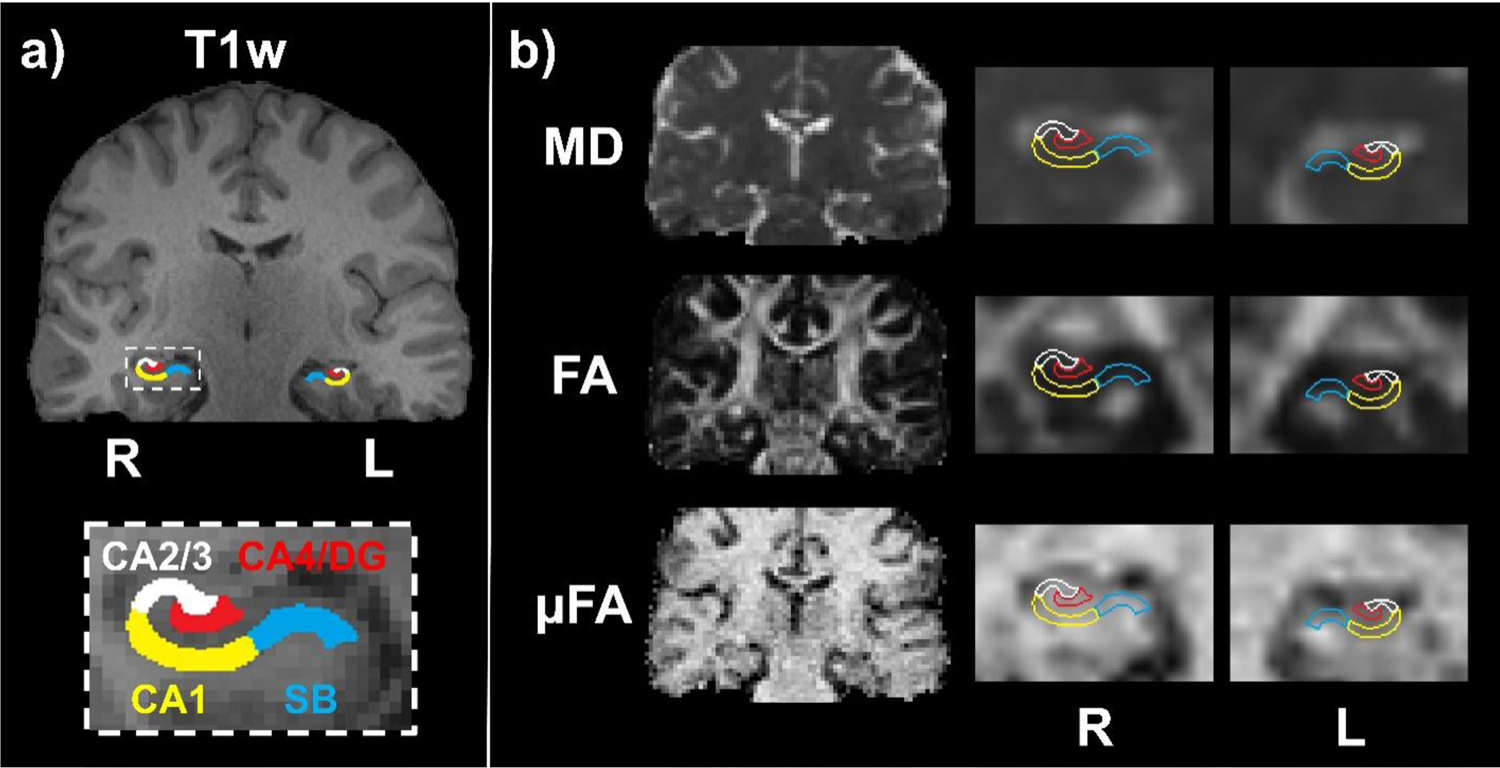
(a) Example T1-weighted coronal image from a drug-resistant TLE patient with unilateral (left) HS evident on structural MRI, with the four hippocampal subregions highlighted. (b) MD, FA, and μFA coronal slices from the same patient before registration to T1-space (left), and after registration to T1-space and interpolation (right) depicting the hippocampal subregions ipsilateral (L) and contralateral (R) to suspected pathology.

### 3.1 Analysis of patients versus controls

Spearman’s rank coefficients relating the metrics measured in the full hippocampus are displayed inFigure 3. In both the TLE and HC cohorts, μFA was correlated with FA in the hippocampus, as expected because both measure diffusion anisotropy, and both anisotropy metrics were negatively correlated with MD. Hippocampal volume did not correlate with any diffusion metrics in the HC cohort, but strongly correlated with μFA in the TLE group.

**Fig. 3.**
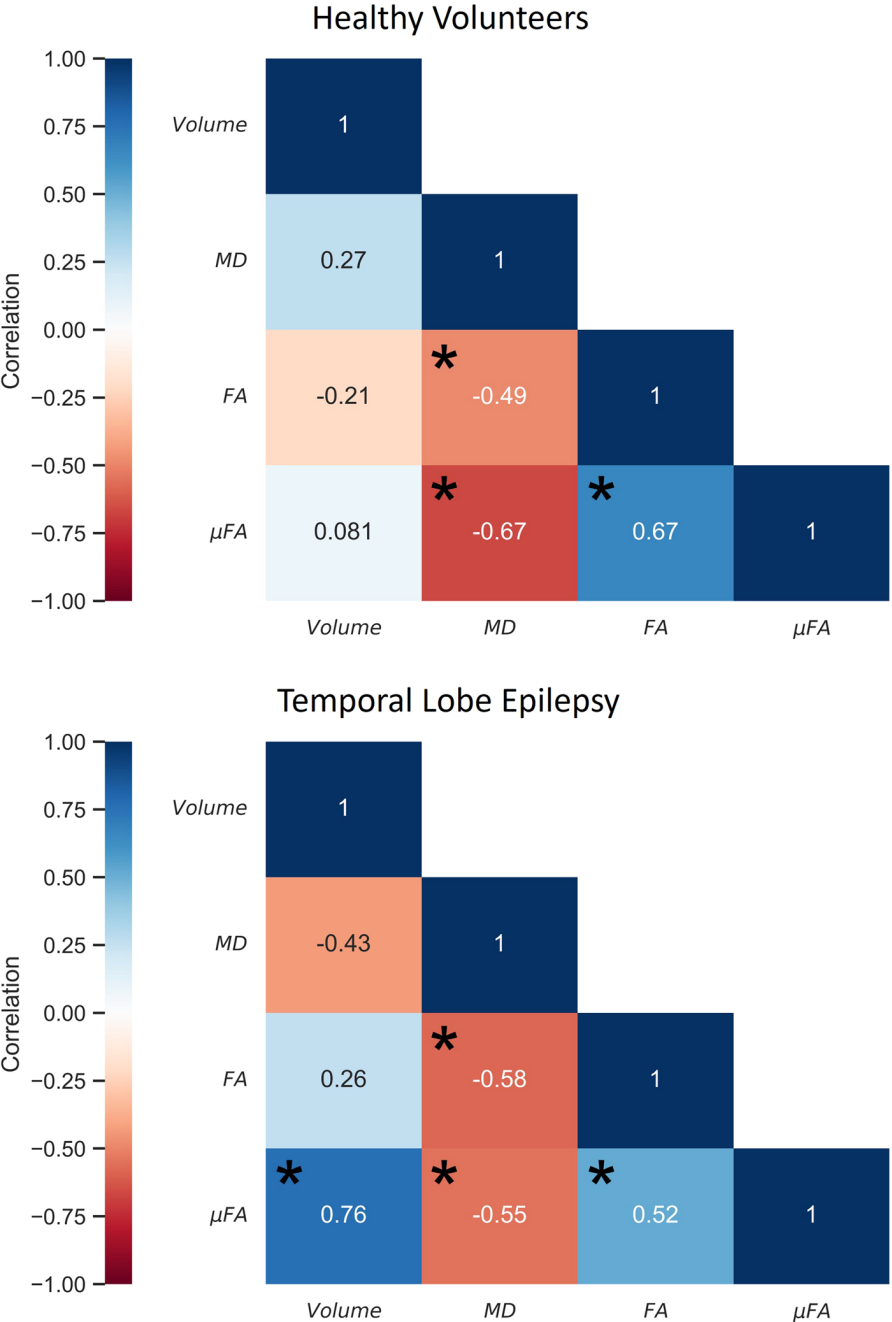
Spearman’s rank correlation matrices comparing hippocampal volume and mean hippocampal measurements of fractional anisotropy (FA), mean diffusivity (MD), and microscopic fractional anisotropy (μFA) in healthy volunteers (top) and in patients with temporal lobe epilepsy (bottom). *p < 0.05.

Mean measurements of volume, MD, FA, and μFA (across both hemispheres) are plotted inFigure 4for the full hippocampus and inFigure 5for each subregion for both cohorts. In the full hippocampus analysis, μFA was the only metric found to be significantly different between the TLE and HC cohorts after correcting for multiple comparisons (p = 0.006), with group mean values of 0.49 ± 0.03 and 0.52 ± 0.03, respectively. In the subregion-specific analysis, μFA in the CA4/DG subregion was found to be significantly different between the cohorts after correcting for multiple comparisons (p = 0.0003), with the TLE group presenting with a lower average μFA (0.47 ± 0.03) than the HC (0.51 ± 0.03).

**Fig. 4.**
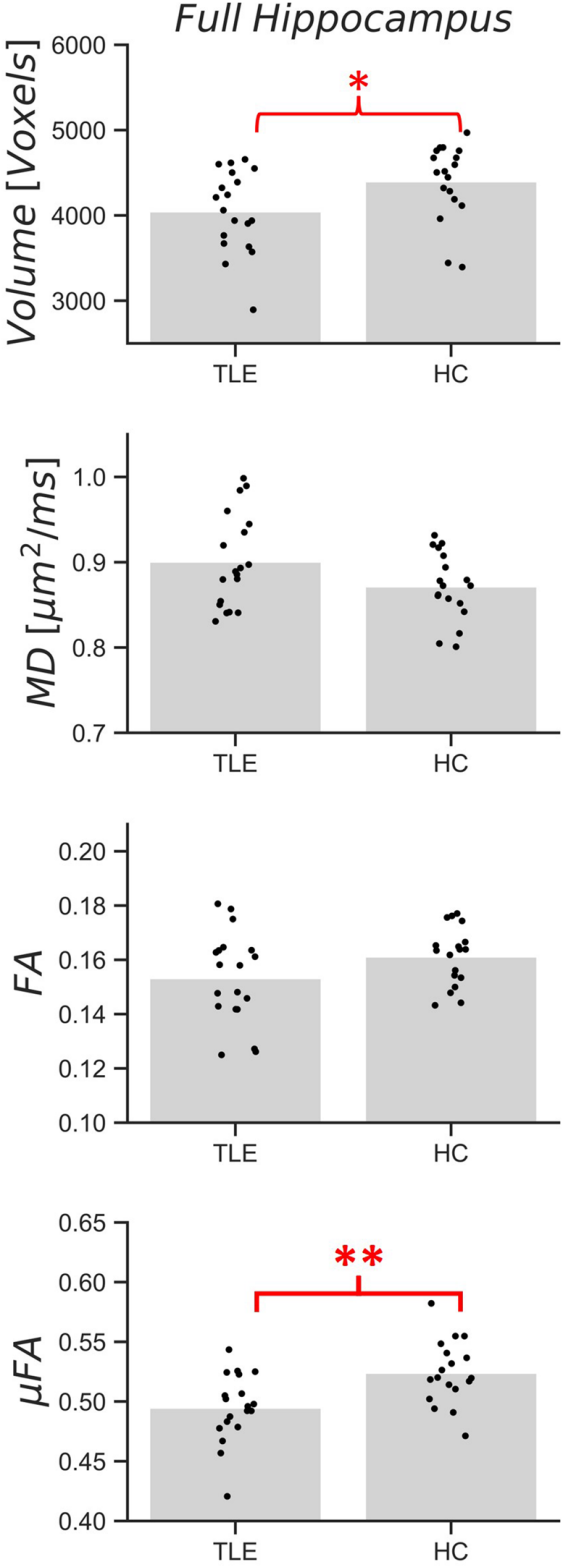
Full hippocampal volume, mean diffusivity (MD), fractional anisotropy (FA), and microscopic fractional anisotropy (μFA) measurements in temporal lobe epilepsy patients (TLE) and healthy volunteers (HC). Each black dot represents the mean across both hemispheres measured in a single patient or volunteer, while the gray bars depict the cohort averages. *p < 0.05, **p < 0.0125.

**Fig. 5.**
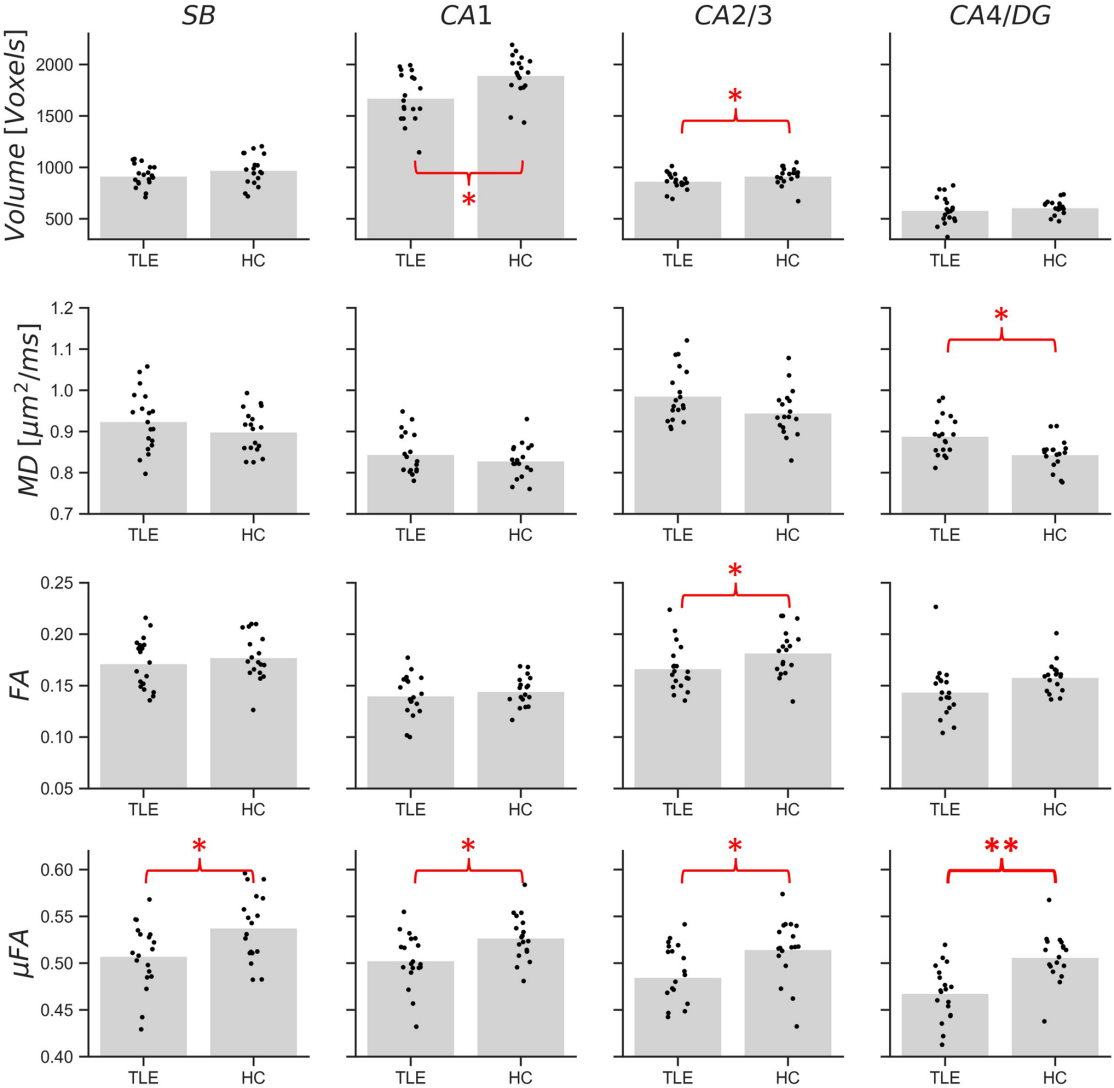
Volume, mean diffusivity (MD), fractional anisotropy (FA), and microscopic fractional anisotropy (μFA) measurements in four hippocampal subregions in temporal lobe epilepsy patients (TLE) and healthy volunteers (HC). Each black dot represents the mean across both hemispheres measured in a single patient or volunteer, while the gray bars depict the cohort averages. *p < 0.05, **p < 0.0031.

ROC curves were computed to index the discriminability of values between patients and healthy controls for the CA4/DG subregion, as it was the only subregion in which a robustly significant difference was observed between the TLE and HC cohorts (Fig. 5). The areas-under-the-curve (AUCs) of volume, MD, FA, and μFA across both hemispheres of the CA4/DG subregion were 0.49, 0.70, 0.69, and 0.81, respectively, while the AUCs of the single-hemisphere measurements of volume, MD, FA, and μFA were 0.64, 0.78, 0.75, and 0.86. Accordingly, single-hemisphere measurements were slightly better at differentiating between cohorts than measurements spanning both hemispheres, and μFA was the best metric for this binary classification. An example ROC curve comparing single hemisphere measurements in the CA4/DG subregion is shown inFigure 6.

**Fig. 6.**
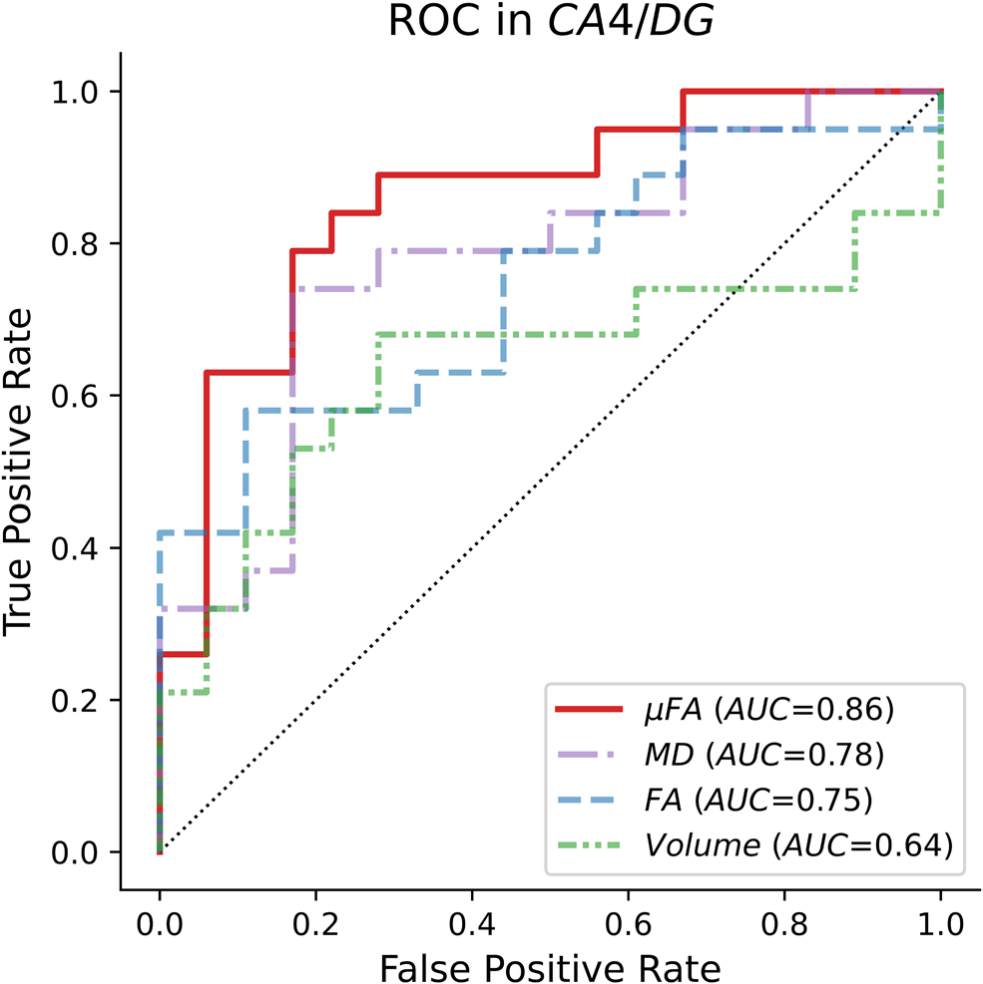
Receiver operating characteristic (ROC) curves depicting the abilities of single-hemisphere CA4/DG subregion measurements to distinguish patients with temporal lobe epilepsy (n = 19) from healthy volunteers (n = 18). The solid red line depicts the ROC curve for microscopic fractional anisotropy (μFA), the purple dash-dotted line depicts the ROC for mean diffusivity (MD), the blue dashed line depicts the ROC for fractional anisotropy (FA), and the green dash-double-dotted line depicts the ROC for subregion volume. The black dotted line depicts a “no skill” classifier with an area-under-the-curve (AUC) of 0.5.

### 3.2 Analysis of ipsilateral–contralateral asymmetry

Measurements of volume, MD, FA, and μFA ipsilateral and contralateral to suspected pathology in the unilateral TLE subgroup (n = 15) are plotted inFigure 7for the full hippocampus and inFigure 8afor each subregion. In the full hippocampus analysis, μFA was the only metric found to be significantly different between the ipsilateral and contralateral hippocampi (p = 0.002), with group mean values of 0.48 ± 0.04 and 0.51 ± 0.02, respectively, and a mean asymmetry index of 4.6%. μFA in the CA4/DG subregion (p = 0.0003) was the only subregion metric found to differ significantly between the ipsilateral and contralateral sides across the cohort, with a lower average μFA in the ipsilateral (0.45 ± 0.04) than in the contralateral side (0.49 ± 0.02), consistent with greater hippocampal pathology ipsilateral to the suspected pathology. The ipsilateral to contralateral asymmetry indices for each measurement in the CA4/DG subregion are depicted inFigure 8b. The mean asymmetry indices in the TLE group were 14.1% (volume), -3.6% (MD), 2.8% (FA), and 9.6% (μFA). In contrast, the mean asymmetry indices in the healthy volunteers were 6.9% (volume), 2.2% (MD), 10.9% (FA), and 3.7% (μFA).

**Fig. 7.**
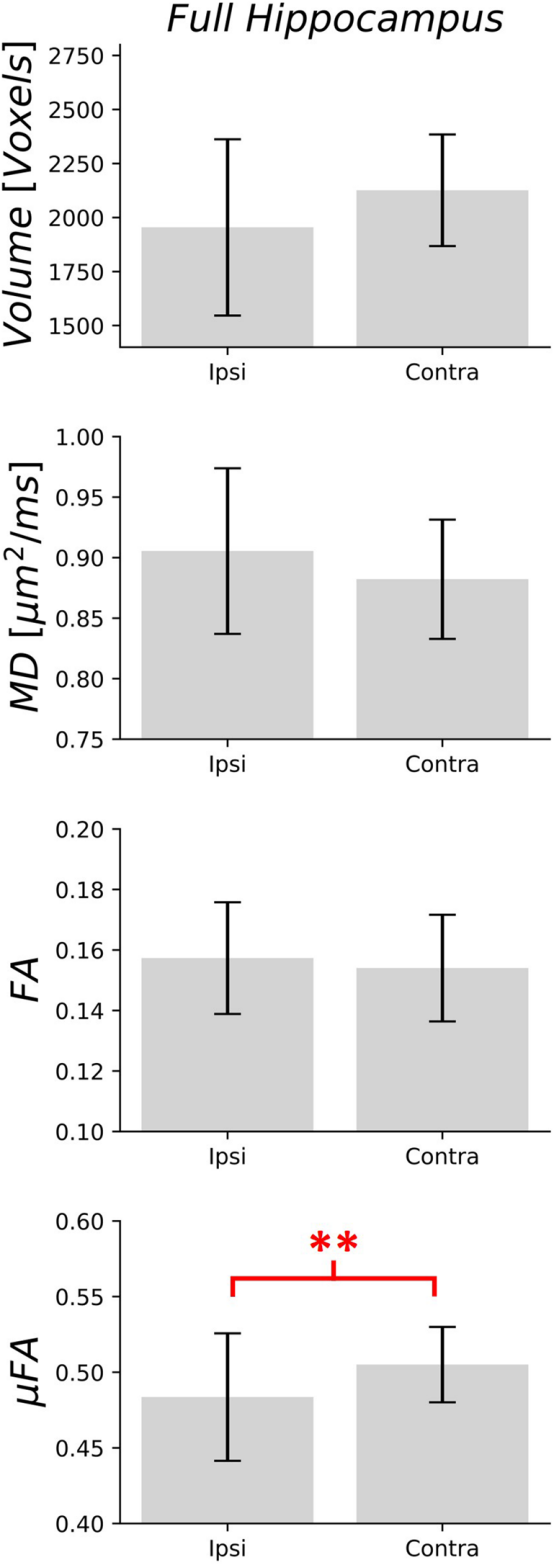
Volume, mean diffusivity (MD), fractional anisotropy (FA), and microscopic fractional anisotropy (μFA) in the full hippocampus in patients with suspected unilateral temporal lobe epilepsy, ipsilateral (Ipsi) and contralateral (Contra) to the suspected seizure focus. The gray bars represent the mean measurements across the entire cohort (n = 15), while the error bars show the standard deviation. **p < 0.0125.

**Fig. 8.**
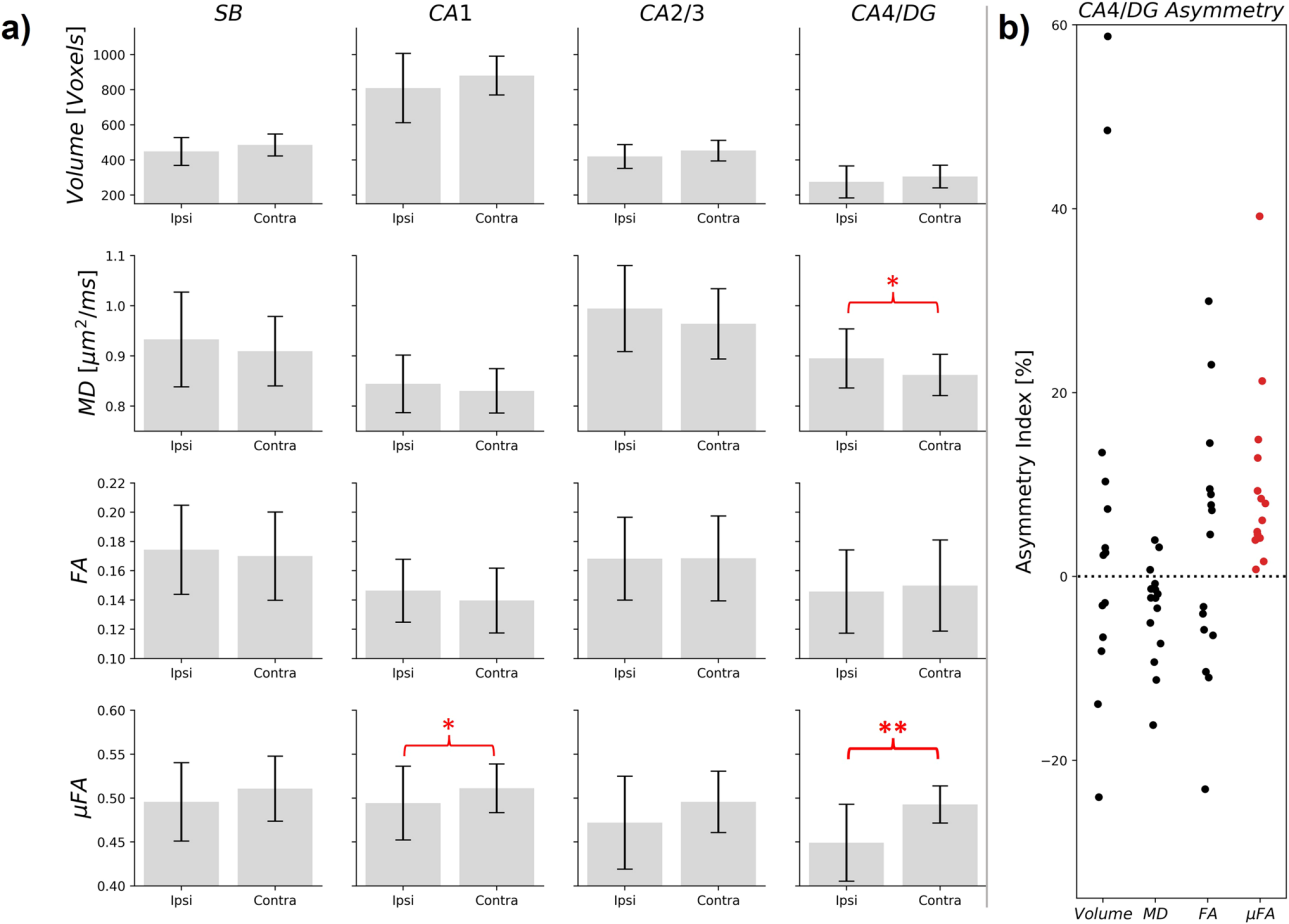
(a) Volume, mean diffusivity (MD), fractional anisotropy (FA), and microscopic fractional anisotropy (μFA) in four hippocampal subregions in patients with suspected unilateral temporal lobe epilepsy (n = 15), ipsilateral (Ipsi) and contralateral (Contra) to the suspected seizure focus. The gray bars represent the mean measurements across the entire cohort, while the error bars show the standard deviation. *p < 0.05, **p < 0.0031. (b) Measurement asymmetry indices in the CA4/DG subregion in suspected unilateral temporal lobe epilepsy patients, defined as the percentage difference between the contralateral and ipsilateral measurements. Each dot represents the ratio measured in a single patient suspected of unilateral disease.

## 4 Discussion

In this study, μFA was found to be sensitive to hippocampal pathology in a mixed cohort of MR-negative and MR-positive TLE patients (Table 1), evidenced by the metric’s ability to differentiate between patients and healthy volunteers, and to correctly lateralize the seizure focus in patients with suspected unilateral disease. This work is the first to apply μFA imaging of the hippocampus or its subregions to the study of TLE.

### 4.1 Patients versus controls

Correlation assessments in the full hippocampus (Fig. 3) revealed that the anisotropy metrics, FA and μFA, were correlated, and that both negatively correlated with MD, in both healthy volunteers and in TLE patients. Volume did not correlate with diffusion metrics in the HC group, but strongly correlated with μFA in the TLE group, potentially reflecting a mutual sensitivity to pathological abnormalities such as HS (Blümcke et al., 2013).

Significant differences between cohorts were observed only for μFA in the full hippocampus. The only subregion with significant differences in any metrics was the CA4/DG subregion, with μFA being lower in TLE patients than in healthy volunteers (Fig. 5), suggesting that this metric may be sensitive to the severe cell loss and gliosis observed in the CA4 and DG regions in patients with ILAE HS types 1 and 3. μFA is thought to be a surrogate marker of axon density and, accordingly, the reductions in μFA may be due to the loss of axons associated with neuron loss (Lampinen et al., 2020). HS type 1 is the most common form of HS, accounting for 60–80% of all cases (Blümcke et al., 2013;Bruton, 1988;Davies et al., 1996). However, if HS type 1 is prevalent among the TLE cohort, one would expect to detect CA1 pathology as well (Blümcke et al., 2013). Modest differences in mean μFA were observed between the TLE and HC cohorts in CA1 but they did not meet the requirements for statistical significance. It is possible that the location of CA1 at the periphery of the hippocampus (compared with CA4/DG which is at the center of the structure) contributed to partial volume effects from extrahippocampal regions containing cerebrospinal fluid (CSF) (Figs. 1and2), or that the rarer HS type 3, in which CA4/DG is affected by pathological abnormalities but CA1 is not, was prevalent in the cohort. Since most of the patients observed in this study had not undergone surgery at the time of writing, HS subtypes could not be confirmed with histology.

Although not statistically significant after correcting for multiple comparisons, the following trends were observed in every subregion: (1) mean MD was greater in TLE than in HC, (2) mean FA was lower in TLE than in HC, and (3) mean μFA was lower in TLE than in HC.

### 4.2 Ipsilateral–contralateral asymmetry

Previous research that used dMRI to study patients with unilateral TLE found ipsilateral MD to be elevated relative to the contralateral side (Assaf et al., 2003;Focke et al., 2008;Kung et al., 2022;Liacu et al., 2010;Treit et al., 2019). In particular,Goubran et al. (2016)observed a strong negative correlation between MD and cell density in CA4/DG, andKung et al. (2022)noted a significantly greater ipsilateral-to-contralateral MD ratio in the DG subregion in TLE patients with probable/definite HS than those with no HS (Kung et al., 2022). Here, CA4/DG MD was found to be greater in TLE patients than in healthy volunteers (p = 0.008), and greater in the ipsilateral side of suspected unilateral TLE patients than the contralateral side (p = 0.005); these observations agree with the literature but did not meet the threshold for statistical significance after correcting for multiple comparisons (p < 0.0031).

Comparisons between ipsilateral and contralateral measurements in suspected unilateral TLE patients revealed that the only significant asymmetries between sides were μFA in the full hippocampus (Fig. 7) and in the CA4/DG subregion (Fig. 8a). Reduced CA4/DG volume in one hemisphere predicted the ipsilateral side in 9 patients (60% accuracy), increased MD predicted the ipsilateral side in 12 patients (80%), and reduced FA predicted in 8 patients (53%). In contrast, μFA in CA4/DG predicted the side consistent with EEG and PET in all 15 patients in the subgroup (100% accuracy), providing strong evidence of the metric’s sensitivity to unilateral pathology. Accordingly, these results, although from a small sample, indicate that μFA has potential to be an early marker of HS. Reduced μFA in one hemisphere of the full hippocampus region correctly predicted the ipsilateral side in 12 patients (80%).

The inability of volume asymmetries to lateralize the epileptogenic zone, despite being a clinical standard for preoperative MRI in TLE (Bernasconi et al., 2019), highlights the need for supplementary imaging techniques in the TLE clinical workflow. In contrast, diffusion metrics are linked to microstructural changes that suggest neuron damage or gliosis, perhaps giving these metrics better specificity to unilateral hippocampal pathology relevant to TLE.

### 4.3 Microscopic fractional anisotropy versus fractional anisotropy

Although FA and μFA both index water diffusion anisotropy on a range from 0 to 1, the two metrics yielded markedly different measurements in the hippocampus. Mean FA values typically fell in the 0.1–0.2 range across all hippocampal subregions and were consistently lower than mean μFA values, which fell in the 0.4–0.6 range. This discrepancy likely results from crossing and fanning fibers in the hippocampus, which attenuate FA measurements but do not affect μFA. The mean values for both anisotropy metrics were consistent with the results ofYoo et al. (2022), in which mean FA and μFA values of 0.2 and 0.47, respectively, were observed in the hippocampi of healthy volunteers. ROC curves comparing the abilities of CA4/DG FA and μFA measurements to classify TLE patients and healthy volunteers based on a threshold value revealed that μFA could differentiate between the cohorts with greater accuracy (Fig. 6), supporting the hypothesis that μFA is a more robust surrogate measure of hippocampal pathology than FA due to its greater specificity in regions with crossing and fanning neurites. Interestingly, both μFA and FA outperformed volume as a marker of pathology, despite volume being a clinical standard for MRI-based detection of HS (Bernasconi et al., 2019).

Although CA4/DG was the only subregion in which a statistically significant asymmetry in μFA was observed in the TLE cohort, mean ipsilateral μFA was lower than contralateral μFA across all four hippocampal subregions in the TLE cohort (Fig. 8a). FA asymmetry in the TLE group was not statistically significant in any of the subregions and was inconsistent across subregions as ipsilateral side FA was greater in SB and CA1 but lower in CA2/3 and CA4/DG relative to the contralateral side. Given that FA values were significantly lower in all subregions relative to μFA values, and that no significant FA asymmetries were observed, it is likely that the sensitivity of FA in detecting hippocampal pathology in TLE is suppressed by its lack of specificity to neuron-fiber microstructure.

### 4.4 Limitations

This preliminary work was limited by the size of both the TLE and HC cohorts, and by the heterogeneity between drug-resistant TLE patients. Although μFA measurements in the suspected unilateral TLE cohort were reliably asymmetric in CA4/DG, future work should include larger sex-matched patient cohorts to validate these findings, investigate potential sex differences, and potentially elucidate asymmetries in other subregions, such as CA1. Notably, the healthy cohort was older and included more females than the drug-resistant TLE cohort. In healthy adults, hippocampal volume and FA have been shown to decrease with age (Solar et al., 2021); hippocampal μFA could follow a similar trend, which would have confounded the TLE versus HC comparisons in this work. That said, the comparisons between contralateral and ipsilateral sides of unilateral patients do not suffer from this limitation.

The first dMRI scan included b-values up to 2000 s/mm^2^, though only the data up to b = 1000 s/mm^2^were used to fit the DTI signal representation. The highest b-shell was excluded because it would introduce biases into the MD and FA metrics due to effects of non-Gaussian diffusion, which become significant at higher b-values (Johansen-Berg & Behrens, 2009). A lower echo time could have been achieved if the b = 2000 s/mm^2^shell was omitted from the scan, which would have resulted in higher signal-to-noise ratio. Furthermore, other acquisition parameters such as multiband factor and resolution were not consistent between the first and second dMRI scans. Thus, there are limitations in the ability to draw conclusions about μFA being more sensitive to TLE pathology than MD and FA from this study. Since MD has been consistently reported to be elevated in the hippocampus in TLE (Goubran et al., 2016;Treit et al., 2019), μFA is likely a complementary measurement, and criteria based on both metrics together may be most appropriate.

The spatial resolutions of the dMRI volumes acquired in this study (1.6–1.8 mm), while acceptable, are suboptimal for visualizing hippocampal subregions (Wisse et al., 2021), so some partial-volume effects near the boundaries between subregions and near CSF likely affected the results. Since the SB, CA1, and CA2/3 subregions encompass the periphery of the hippocampus, they could be more susceptible to partial volumes of extrahippocampal brain tissue or CSF; contrarily, the CA4/DG region lies in the center of the hippocampus and would only be affected by partial volumes of other hippocampal subregions. The significant μFA asymmetry in CA4/DG demonstrates potential for lateralization of the epileptic zone in TLE and demonstrates the increased utility of μFA over FA in studying the hippocampus. In future work, the spatial resolution could be improved at the expense of increased scan duration; the μFA protocol used in this study required 6 min to achieve 1.8 mm resolution, butYoo et al. (2022)demonstrated a μFA protocol with 1.5 mm isotropic resolution that could be acquired in 15 min. To counter the increased scan time needed for higher resolution, the field-of-view and number of slices could be reduced to capture a smaller subvolume of the brain containing the hippocampus. Additionally, techniques to mitigate CSF partial-volume effects, such as a recently proposed free water elimination μFA protocol (Arezza et al., 2023), could be employed to increase specificity to pathology.

Though scalp EEG is highly specific for lateralizing the ipsilateral seizure onset (Javidan, 2012), the more invasive intracranial EEG is the gold standard for lateralizing MR-negative patients, and only histopathological assessment of excised tissue can confirm the presence of HS with absolute certainty. Furthermore, the suspected unilateral TLE subgroup could have contained patients with bilateral disease or those who were incorrectly lateralized by EEG. Thus while the agreement observed between μFA and scalp EEG in lateralizing pathology is promising, results should be interpreted with caution due to the absence of ground truth lateralization for most patients (seeSection 4.6).

A diagnostic biomarker should have a large effect size to be useful at a single subject level, which helped motivate the rather conservative Bonferroni corrections used here to compare measurements between cohorts and to detect asymmetries (Armstrong, 2014). While some significant trends may have been undetected, μFA in the CA4/DG significantly differed between TLE and HC cohorts, and μFA in CA4/DG differed significantly between the ipsilateral and contralateral hemispheres in the suspected unilateral TLE cohort even with this stringent correction. Most notably, μFA asymmetry in CA4/DG correctly lateralized every patient in the suspected unilateral TLE cohort, which included nine patients in whom pathology was not evident on structural MRI.

Several methods to measure anisotropy independent of orientation dispersion have been proposed and demonstrated in studies, including techniques based on double diffusion encoding (Jespersen et al., 2013) and biophysical modeling (Kaden et al., 2016) in addition to the spherical tensor encoding-based technique used in this work. Double diffusion encoding and the spherical tensor encoding used here follow the same theoretical underpinnings, so it is expected that they would both give similar results. The approach byKaden et al. (2016), which does not use b-tensor encoding, is intended for use in white matter and uses biophysical modeling with assumptions about the tissue (e.g., glial cells, soma, and extracellular space are not differentiated from each other). Accordingly, this approach may not be applicable in the gray matter of the hippocampus.

### 4.5 MR-negative versus MR-positive temporal lobe epilepsy

The TLE cohort observed in this study included both MR-positive and MR-negative patients. To assess whether the significant results discussed in this work were driven by the MR-positive patients who presented with more advanced pathology, we investigated removing the MR-positive patients from the suspected unilateral TLE cohort. In this MR-negative suspected unilateral subgroup (n = 9, 5 males to 4 females, mean age = 29.2 ± 5.6), it was found that the difference between ipsilateral and contralateral μFA in the CA4/DG region was still significant after correcting for multiple comparisons (p = 0.002). The average asymmetry index in this reduced cohort was 4.1% (compared with 9.6% when MR-positive patients were included). No other metric or region combination, including full hippocampus μFA, showed significant asymmetry in this cohort.

### 4.6 Postsurgical assessment

As of the time of writing, four TLE patients (IDs 1–4 inTable 1) have undergone anterior temporal lobectomy procedures with full amygdalohippocampectomy, all on the left side, and the results of pathological assessment are available for two of the patients (IDs 1 and 4). Prior to surgery, patients 2 and 3 presented with left-sided HS detectable in MRI, but patients 1 and 4 were MR-negative. While all four patients presented with lower ipsilateral μFA in CA4/DG in preoperative imaging, the two MR-positive patients exhibited much greater hemispheric asymmetry (>12%) than the MR-negative patients 1 (1.6%) and 4 (4.1%). Postsurgical pathological assessment revealed no evidence of HS in the tissue excised from patient 1, but HS type 1 in patient 4. While these results show promising correspondence between μFA asymmetry and HS, more subjects are needed to investigate the relationship between histopathology and MRI metrics like μFA.

## 5 Conclusions

This study demonstrated that the combination of hippocampal subregion segmentation with μFA imaging may be helpful for lateralizing and localizing the epileptogenic zone in patients with unilateral TLE. The μFA protocol used in this work is clinically feasible because the scan was performed at a clinical field strength of 3T and only required 6 min of total scan time. Assuming the poorer surgical outcomes experienced by patients with MR-negative TLE are in part due to poorer identification of the seizure focus, then dMRI techniques that can complement the current techniques for lateralizing and localizing the seizure focus may lead to improved surgical outcomes in these patients. Additionally, μFA can provide a useful metric for basic science research that aims to better understand TLE at the population level due to its strong specificity to microstructure.

## Data Availability

Code for our implementation of microscopic fractional anisotropy fitting is available athttps://gitlab.com/cfmm/matlab/matmri.
